# A novel *Bacillus ligniniphilus* catechol 2,3-dioxygenase shows unique substrate preference and metal requirement

**DOI:** 10.1038/s41598-021-03144-8

**Published:** 2021-12-14

**Authors:** Peter Adewale, Alice Lang, Fang Huang, Daochen Zhu, Jianzhong Sun, Michael Ngadi, Trent Chunzhong Yang

**Affiliations:** 1 Bioprocessing and Biocatalysis Team, Aquatic and Crop Resource Development Research Centre, National Research Council Canada, 100 Sussex Drive, Ottawa, ON K1A 0R6 Canada; 2 School of Environment and Safety Engineering, Jiangsu University, Zhenjiang, Jiangsu China; 3 Bioresource Engineering Department, McGill University, 21111 Lakeshore Rd., Ste-Anne-de-Bellevue, QC H9X 3V9 Canada

**Keywords:** Biochemistry, Biotechnology, Molecular biology

## Abstract

Identification of novel enzymes from lignin degrading microorganisms will help to develop biotechnologies for biomass valorization and aromatic hydrocarbons degradation. *Bacillus ligniniphilus* L1 grows with alkaline lignin as the single carbon source and is a great candidate for ligninolytic enzyme identification. The first dioxygenase from strain L1 was heterologously expressed, purified, and characterized with an optimal temperature and pH of 32.5 °C and 7.4, respectively. It showed the highest activity with 3-ethylcatechol and significant activities with other substrates in the decreasing order of 3-ethylcatechol > 3-methylcatechol > 3-isopropyl catechol > 2, 3-dihydroxybiphenyl > 4-methylcatechol > catechol. It did not show activities against other tested substrates with similar structures. Most reported catechol 2,3-dioxygenases (C23Os) are Fe^2+^-dependent whereas *Bacillus ligniniphilus* catechol 2,3-dioxygenase (BLC23O) is more Mn^2+^- dependent. At 1 mM, Mn^2+^ led to 230-fold activity increase and Fe^2+^ led to 22-fold increase. Sequence comparison and phylogenetic analyses suggested that BL23O is different from other Mn-dependent enzymes and uniquely grouped with an uncharacterized vicinal oxygen chelate (VOC) family protein from *Paenibacillus apiaries*. Gel filtration analysis showed that BLC23O is a monomer under native condition. This is the first report of a C23O from *Bacillus ligniniphilus* L1 with unique substrate preference, metal-dependency, and monomeric structure.

## Introduction

Many microorganisms have adaptively grown by developing catabolic pathways to utilize the available compounds in their natural environments, such as forest, soil, water, and sediments. The metabolic activities of these microorganisms within their habitats have provided useful information on the formation pathways of many value-added products and their intermediate routes. The microbial degradation of large aromatic polymers such as lignin and its derivatives has gained extensive attention as an essential strategy for future bio-refineries, and circular bio-economy^1–3^. More so, the biological conversion of lignin and its derivatives has been an area of interest for many researchers due to the product specificity and mild conditions of the conversion processes^4^. The utilization of fungal systems, such as extracellular peroxidase and laccase from white and brown rot fungi^5–7^, on lignin derivatives degradation has been well established. Commercial grade oxidoreductases, including manganese peroxidase, laccase, lignin peroxidase, and versatile peroxidase secreted by brown and white-rot fungi have been widely utilized^8,9^. However, biodegradation of lignin and its derivatives by bacterial enzymes has not been extensively reported as fungal enzymes.

Nevertheless, many reports have suggested the importance of bacteria in lignin and its derivatives degradation to value-added metabolites^10,11^. Several bacterial enzymes from the class of actinomycetes such as *Amycolatopsis* sp. ATCC 39116^12^, *Rhodococcus opacus*^13,14^, *Rhodococcus* sp. YHY01^15^, and *proteobacteria phylum* such as *Pseudomonas putida* NX-1^16^, *Pseudomonas putida* KT2440^17,18^, and *Cupriavidus necator* JMP134^19,20^ have been explored. In recent years, numerous bacterial strains with metabolic capabilities to utilize lignin and its derivatives have been isolated from the genus *Bacillus*, e.g. *Bacillus pumilus*^21,22^, *Bacillus atrophaeus* ^23,24^. Most of these organisms have been tested to possess pathways for the catabolism of lignin and its aromatic derivatives such as catechol, alkyl catechols, vanillin, and guaiacol as their sole carbon source for growth.

Zhu et al.^25^ identified an alkaline lignin degrading bacterium (*Bacillus ligniniphilus* L1) isolated from sediment samples at a benthal depth of 3,000 m in the South China Sea. Recently, *Bacillus ligniniphilus* has been proposed to be classified as *Alkalihalobacillus ligniniphilus*^26^ and the genome database is under *Alkalihalobacillus ligniniphilus* strain L1. Strain L1 grows with alkaline lignin as the single carbon source. Three pathways for its lignin degradation are suggested, namely, the gentisate pathway, the benzoic acid pathway, and the β-ketoadipate pathway (protocatechuate). Fifteen aromatic compounds were identified during the strain L1 degradation process of alkaline lignin. After lignin degradation into phenolic monomers, the very last step in lignin metabolic pathways are catalyzed by dioxygenases that open the aromatic ring of catechol to a linear structure. Putative open reading frames (orf) encoding enzymes involved in lignin degradation of strain L1 were predicted based on genome sequence including genes coding for dioxygenases^25^.

Dioxygenases are classified into two families, intradiol and extradiol dioxygenases, that catalyze the oxidative cleavage of catechol^27,28^. The intradiol dioxygenases cleave the C–C bond between the phenolic hydroxy groups to yield muconic acid as the product^29,30^ whereas the extradiol dioxygenases (EDOs) cleave the C–C bond adjacent to the phenolic hydroxy groups to yield 2-hydroxymuconaldehyde as the product^31^. These enzymes require metals as cofactors, predominantly iron (Fe^3+^, Fe^2+^)^32,33^. Among the extradiol dioxygenase subfamily are catechol 2,3-dioxygenases that catalyze the ring cleavage of catechol and its alkyl derivatives. These enzymes have been found in both Gram-negative (*Pseudomonas, Sphingomonas, Acinetobacter, Burkholderia, Stenotrophomonas*)^18,34,35^ and Gram-positive (*Rhodococcus, Bacillus*) bacterial strains^10,36^.

Even though many orfs encoding lignin degradation enzymes including three for catechol 2,3-dioxygenases (C23O) were predicted in strain L1, so far, only one laccase enzyme has been characterized^37^. None of the putative dioxygenase genes has been confirmed with enzyme activity and characterized. Since strain L1 can grow with alkaline lignin as its sole carbon source, it is predicted to have powerful enzymatic systems to break down lignin into monomer and linear structures.

Compared with the fungal lignin-degrading enzymes, the overall enzymology for bacterial lignin degradation is poorly understood, yet there are indications that bacteria use similar types of extracellular lignin-degrading enzymes to fungi. For example, aromatic degradation pathways in soil bacteria have been extensively studied. As in fungi, lignin polymer degradation is followed by metabolisms of aromatic compounds through aromatic ring cleavage enzymes such as catechol 2,3-dioxygenase and many oxidative pathways involving ortho-cleavage and meta-cleavage of catecholic intermediates have been elucidated^38–40^. Whole genome sequence suggests that strain L1 may utilize 3 pathways for lignin degradation and three orfs encoding potential C23Os are identified^25^. To benefit from the understanding of soil bacterial aromatic degradation pathways, we chose to study C23Os as a starting point to explore strain L1 lignin degradation processes. We reasoned identification of novel C23O may help to eventually understand if strain L1 utilize unique mechanism to grow with alkaline lignin as a sole carbon source.

Among the three identified strain L1 C23O orfs, gm_orf 726 encoded a protein of 283 aa (BLC23O, accession number: WP_017726464.1), gm_orf3186 encoded a 319 aa protein (BLC23O-2, accession number: WP_017728832.1) and gm_orf2069 encoded a 326 aa protein (BLC23O-3, accession number: WP_017727756.1). BLC23O-2 and BLC23O-3 not only have similar size but also significant sequence identity (69%) whereas BLC23O has only 20% and 19% sequence identities with BLC23O-2 and -3. Blast searching of protein databases with BLC23O-2 and -3 amino acid sequences can easily identify many characterized proteins with > 70% sequence identities including 11 and 6 sequences with > 80% identities for BLC23O-2 and -3 respectively. In contrast, with BLC23O, no protein database sequence was identified with > 60% identity. It is highly questionable if BLC23O has any enzyme activity and if so, there could be a better chance to identify a novel enzyme activity than the other two candidates. Therefore, the shortest protein, BLC23O, was chosen as the first candidate. BLC23O was expressed in *E.coli*, purified, and its activities were characterized. To the best of our knowledge, this is the first work to characterize an active dioxygenase enzyme (BLC23O) from *Bacillus ligniniphilus* L1.

## Materials and methods

### Materials

The electrophoresis markers and Bradford Protein Assay (BPA) reagent for protein quantification were obtained from Bio-Rad Labs. All other chemicals and reagents were of analytical-reagent grade and purchased from Millipore Sigma.

### Cloning and overexpression of BLC23O gene

Putative BLC23O encoding gene (gm_orf 726) as reported by Zhu et al.^25^ was codon optimized for *E. coli* expression (Fig. SI1), synthesized and cloned into PET28b + vector by ligating into the *Nde* I and *Xho* I restriction sites (Bio Basic Inc). The expressed protein contains N-terminal 6 × His tag followed by the thrombin cleavage site for purification. The plasmid was transformed into chemically competent *E. coli* BL21 (DE3) cells. The transformed bacterial cell colonies were used to inoculate 5 mL of LB media containing kanamycin (30 µg/mL) and grown at 37 °C overnight. The next day, 1 mL of the culture was used to inoculate 100 mL of LB media with 30 µg/mL kanamycin at 37 °C until the optical density (OD) at 600 nm reached 0.4 to 0.6. Expression of the recombinant protein was induced with 0.2 mM Isopropylthio-β-galactoside (IPTG, Sigma-Aldrich) and grown at 16 °C for 19 h. The cells were harvested by centrifugation at 4 °C, 3000 × g for 20 min. The cell pellets were frozen and stored at -20 °C.

### BLC23O purification

The cell pellets were thawed on ice and then suspended in the lysis buffer containing 50 mM NaH_2_PO_4_, 300 mM NaCl and 10 mM imidazole (pH 8.0). To the suspension, 0.1 mM phenylmethylsulfonyl fluoride (PMSF), 3 U/mL Benzonase (Sigma-Aldrich, E1014) and 1 mg/mL lysozyme were added, and the mixture was incubated at 4 °C for 30 min with rotation. The cells were disrupted by sonication at 20 secs intervals with 30 s cooling periods on an ice bath at 30% maximal power until clear lysate was achieved (Sonicator 3000, Misonix Inc.). The lysate was clarified by centrifugation at 3,000 × g at 4 °C for 20 min.

The recombinant enzyme was isolated from the crude extract supernatant by 6 × His-tag Ni–NTA agarose affinity column (Qiagen) chromatography following the manufacturer’s instructions using a 50 mM NaH_2_PO_4_, 300 mM NaCl,10 mM imidazole (pH 8.0) as the binding solution, a 20 mM imidazole (pH 8.0) wash solution and eluted with a 250 mM imidazole (pH 8.0) solution. The eluted fractions containing BLC23O were pooled, concentrated, and the buffer was changed with 10 k molecular weight (MW) cut-off centrifugal filters (Amicon Ultra) to 0.1 M Tris–HCl (pH 7.4) buffer. The extract was subsequently purified with size-exclusion chromatography (SEC) through a Hiload 16/60 Superdex 200 gel filtration column (GE Healthcare Life Sciences) using an AKTA fast protein liquid chromatography (FPLC) system (Pharmacia Amersham Biotech) for kinetics study. The column was equilibrated and eluted with 0.1 M Tris–HCl, 150 mM NaCl buffer (pH 7.4) at a flow rate of 1.0 mL/min and room temperature. The eluted peaks were monitored at 280 nm. 1 mL fractions were collected. The purified enzyme was kept at -20 °C with 30% glycerol and stable during the experimental period. The purified enzyme was analyzed with 12% SDS-PAGE gels, and quantified with BPA assay and Bovine serum album (BSA) as the standard.

To exam the oligomeric state of BLC23O, the purified enzyme was analyzed by gel filtration under native condition using Hiload 16/60 Superdex 200 column (GE Healthcare Life Sciences) with an AKTA FPLC system (Pharmacia Amersham Biotech) under the conditions described above for BLC23O FPLC purification.

### Enzyme assay

The assay mixture contained 1 mM 3-methylcatechol (20 µL of 50 mM stock made in ddH_2_O), 50 µL purified protein extract, and 930 µL of phosphate buffer pH 7.5 (50 mM) to bring the total reaction volume to 1 ml. Protein content was quantified using the BPA assay kit. The mixture was vortexed briefly to homogenize its composition. Then, 250 µL of each reaction mixture was transferred into a 96-well plate in triplicate for enzyme activity. The product formation was monitored for 2 h, every 10 min interval at 40 °C using a Spectrophotometer (SpectraMax M5, Molecular Devices, San Jose, CA, USA) at the maximum wavelength corresponding to the formation of the product from the substrates. The control contained the same compositions as the enzyme assay excluding the protein extract and was processed under the same conditions. The assay was developed with reference to published methods^41–43^. One unit of enzyme activity (U) was defined as the amount of enzyme that converts one μmole of substrate per min. Specific activity was defined as μmol of product formed per minute per mg of total protein. For the cleavage product out of different substrates, the specific wavelength, λmax, and the molar extinction coefficients (ε) used under the assay condition were listed in “section Substrate preference of BLC23O from strain L1”. Specific enzyme activity (μmol/min/mg) was determined by Eq. 1:

1 $$\frac{{OD_{\lambda } \left( {absorbance} \right)}}{{time \left( {\min } \right)}} \div \varepsilon ({\text{cm}}^{ - 1} \,{\text{M}}^{ - 1} ) \times reaction\,volume \,({\text{L}}) \div protein\,({\text{mg}}) \times 10^{6}$$

### Effect of temperature and pH on BLC23O activity

Optimum temperature (Topt) and pH (pHopt) for BLC23O were determined by measuring the activity under the temperatures of 25, 30, 32.5, 35, 40 and 45 °C at pH 7.4 and the pHs of 6.0, 7.0, 7.2, 7.4, 7.6, 7.8, 8.0 and 8.5 at 32.5 °C, respectively. The reaction mixture contains 0.1 M Tris–HCl buffer, 50 µg/mL purified enzyme extract, 1 mM of 3-methylcatechol (substrate), and 0.1 mM MnCl_2_·4H_2_O. The 250 µL of reactions were carried out in a 96-well plate and absorbances were  measured with a spectrophotometer (SpectraMax M5, Molecular Devices, San Jose, CA, USA) at 388 nm in triplicate. The absorbance readings were measured for 20 min at 30 s intervals. The relative activity was calculated based on OD change compared to maximum (100%) activity at pH 7.4^44^.

The half-life of enzyme activity was measured by performing the enzyme reaction assay after incubating the enzyme at different temperatures (40, 52, 54, and 56 °C) for different lengths of time (0, 5, 10, and 15 min). Aliquots were taken from the heated enzyme solution and immediately cooled on ice. A 50 µg/mL enzyme was combined with 1 mM 3-methylcatechol, 0.1 mM MnCl_2_·4H_2_O in 0.1 M Tris–HCl buffer (pH 7.4) to start the reaction. The assay was conducted at 32.5 °C for 20 min at 30 s intervals in triplicate. The amount of product generated was evaluated by measuring the absorption at 388 nm to determine enzyme specific activity and changes over time. A first-order deactivation rate constant (k_d_) was determined by fitting the experimental data to an exponential model using MS Excel. The half-life (t_1/2_) of BLC23O at 40, 52, 54, and 56 °C was calculated using Eq. 2:

2 $$t_{1/2} = \frac{\ln 2}{{k_{d} }}$$

### Substrate preference of BLC23O from strain L1

BLC23O substrate preference was examined with twelve catecholic compounds, namely catechol, 3-methylcatechol, 3-ethylcatechol, 3-isopropylcatechol, 4-methylcatechol, 4-chlorocatechol, 2, 3-dihydroxybiphenyl, 3-fluorocatechol, protocatechuic acid, 2, 5-dihydroxybenzoic acid, pyrogallol, and 1, 2-dihydroxynapthalene using the spectrophotometric method^44^. These compounds were selected based on their varying substituents and similarity to intermediate compounds observed in various lignin degradation pathways. The spectra were obtained at 32.5 °C in 0.1 M Tris–HCl (pH 7.4) buffer that contains 0.1 mM MnCl_2_·4H_2_O, 90 µg/mL enzyme, and 1 mM each of the aromatic compounds as a substrate. 1, 2-dihydroxynaphthalene was dissolved in 10% tetrahydrofuran (THF). 2, 3-dihydroxybiphenyl was dissolved in 5.7% ethanol and 3-isopropylcatechol and 3-ethylcatechol were dissolved in 4.8% ethanol. The UV–vis (200–550 nm) spectrum of the reaction mixture and the control were captured at 2 or 5 nm steps and a series of time points from 0 to 30 min. Each curve represents the mean of triplicate measurements. In general, the noticeable appearance and increase of a peak in the UV spectrum over time corresponding to wavelength of the expected cleavage product indicated that a specific reaction occurred. For substrates without product UV wavelength data, screening for any changes in spectrum or peaks were performed to detect cleavage product formation. No peak or noticeable change in spectra indicated no cleavage product formation.

To obtain specific activities of BLC23O against active substrates, assays were performed in 0.1 M Tris–HCl (pH 7.4) buffer with 1 mM substrate, 60 µg/mL BLC23O and 0.1 mM MnCl_2_·4H_2_O. Absorbance was measured at the wavelength corresponding to expected cleavage product over 20 min at 30 section intervals. Specific activities were calculated from the initial linear slopes. The numbers are averages from triplicate experiments. All numbers are expressed as percentage specific activities with catechol set as 100% and compared with literature reported C23O activity. The molar extinction coefficient and wavelength employed for the product of catechol were 33,400 M^−1^ cm^−1^ and 375 nm (pH 7.6), for 3-methylcatechol were 13,800 M^−1^ cm^−1^ and 388 nm (pH 7.6), for 3-ethylcatechol were 10,900 M^−1^ cm^−1^ and 390 nm (pH 7.5), for 4-methylcatechol were 28,100 M^−1^ cm^−1^ and 382 nm (pH 7.6), for 4-chlorocatechol were 39,600 M^−1^ cm^−1^ and 379 nm (pH 7.5), for 2,3-Dihydroxybiphenyl were 13,200 M^−1^ cm^−1^ and 434 nm (pH 7.5), and for 3-isopropylcatechol were 18,500 M^−1^ cm^−1^ and 393 nm (pH 7.5)^44–49^.

### Effect of metal ions on BLC23O activity

The metal effect testing process was designed based on published literatures^28,47^. The effects of six metal ions were investigated by adding metal compounds into the BLC23O solution at two final concentrations of 0.1 mM and 1.0 mM. The metal compounds used were CuSO_4_, FeSO_4_·7H_2_O, FeCl_3_, MgCl_2_·6H_2_O, KCl, and MnCl_2_·4H_2_O. The reaction mixtures consisted of a specific volume of 0.1 M Tris–HCl buffer (pH 7.4), selected concentrations of metal solutions, purified protein extract, and 1 mM 3-methylcatechol as the substrate^47^. The enzyme activity was spectrophotometrically measured by following the increase in absorbance at 388 nm over 30 min at 2 min intervals. The linear increase in the absorbance versus time curve was used to calculate the slope of each reaction and control mixture. For each reaction, the absorbance change of the control (without enzyme) was subtracted to obtain a final value corresponding to activity. The relative activity of each reaction was determined as a percent in comparison to the control reaction with no addition of extra metal (100% activity). Each value was recorded as an average of triplicate tests.

### Enzyme reaction kinetic modelling

The catalytic (*k*_cat_) and Michaelis–Menten (*K*_m_) constants of BLC23O were determined for the two most preferred substrates (3-methylcatechol and 3-ethylcatechol) as shown in Table 2. The reaction mixture was incubated at 32.5 °C in 0.1 M Tris–HCl buffer (pH 7.4) containing 0.1 mM MnCl_2_·4H_2_O. 40 µg/mL BLC23O was used for 3-methylcatechol kinetic assay and 20 µg/mL enzyme was used for 3-ethylcatechol reactions. The reactions were initiated by adding various substrate concentrations of 0.2–1.2 mM 3-methylcatechol or 0.05–1.0 mM 3-ethylcatechol. The activity of BLC23O was measured by the increase of cleavage product formation at OD 388 nm (3-methylcatechol) or 390 nm (3-ethylcatechol) every 30 s for 30 min in a 96 well UV-Star microplate (Greiner Bio-one) using a spectrophotometer (SpectraMax M5, Molecular Devices, San Jose, CA, USA)^45^. The initial linear slopes were used to determine the kinetic parameters of BLC23O. The extinction coefficient used for each substrate has been listed in “section Substrate preference of BLC23O from strain L1”. The experimental data was fitted using the nonlinear regression model and processed with Graph pad 8 software to determine V_max_, *K*_m_, *k*_cat_, and *k*_cat_/*K*_m_. The parameter results were obtained from the fitting of triplicate sets of data and presented as mean and standard deviation.

### Statistical analysis

One-way ANOVA design was used for the statistical analysis of the data using JMP Pro (Statistical Analysis Systems, version 13.2, SAS Institute Inc., Cary, NC, USA). All tests were performed in three independent replicates. Mean comparisons of each of the data points and their significant differences were analyzed by Tukey’s studentized range test denoted as means ± standard errors.

## Results and discussion

### Cloning, overexpression, and purification of functional BLC23O

Based on sequence analysis, the BLC23O enzyme contains 283 aa with a calculated molecular mass of 31,825 Da and theoretical pI of 5.5. The synthesized gene encoding BLC23O was cloned into the *E. coli* expression vector pET-28b (+) and fused to the vector carried sequences coding for the His-tag and thrombin cleavage site at N-terminal. The fused full-length protein is 303 aa with an estimated molecular mass of 33,988 Da and theoretical pI of 5.93. For efficient soluble BLC23O enzyme expression in the transformed *E. coli* strain, extensive variations of culturing and inducing conditions were tested. The impacts of growth temperature, time, IPTG concentration and induction time were evaluated. SDS-PAGE analysis showed that a significant band occurred at expected size of ~ 34 kDa (Fig. SI 2). Culturing at 37 °C with 1 mM IPTG induction led to most proteins expressed in insoluble form. Decreased IPTG concentration led to an increased soluble fraction. Under 16 °C, the majority of expressed proteins were soluble with different IPTG concentrations from 0.01 to 1 mM. Based on these tests, the selected inducing and culturing conditions for BLC23O soluble expression were: 0.2 mM IPTG with overnight growth at 16 °C.

**Figure 1:**
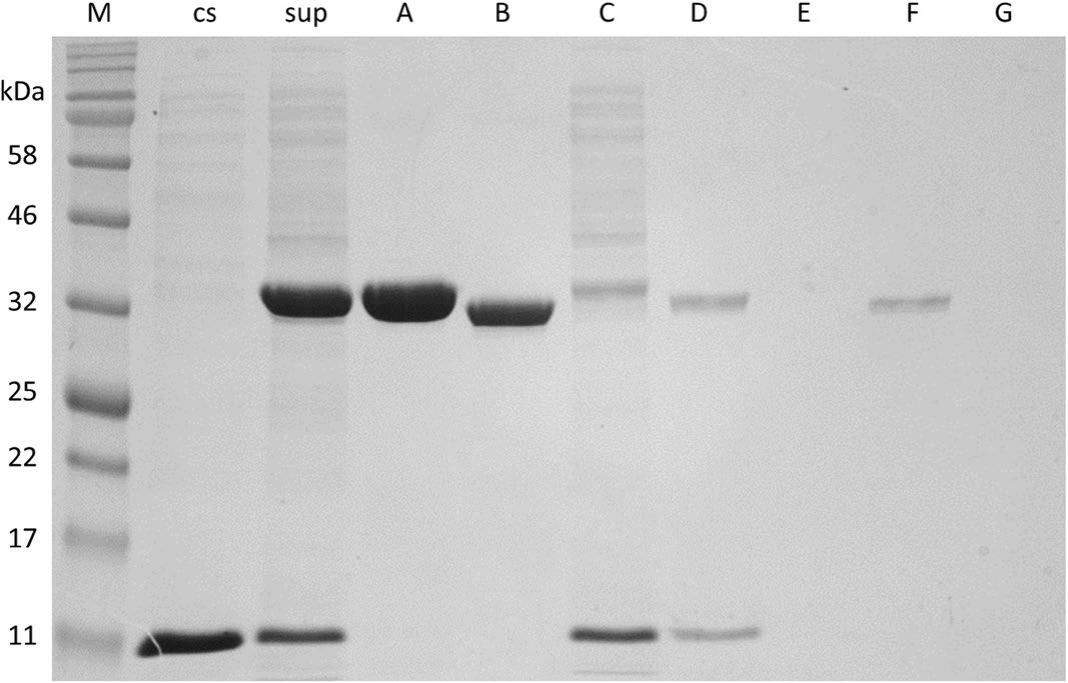
SDS-PAGE analysis of samples during purification of BL C23O. cs, control sample from transformant of empty vector, sup, cell lysate supernatant of C23O transformant, A, his-tag purified sample, B, purified sample after thrombin cleavage; His-tag purification waste: C = initial eluate, D = wash solution, E = buffer change solution; Thrombin cleavage waste: F = wash solution, G = elution solution.

Following established culturing conditions for soluble BLC23O enzyme expression, transformed *E. coli* cells were grown up and induced with IPTG. The protein was isolated from the crude extract by 6 × His-tag Ni–NTA affinity column chromatography. Further purification was achieved using thrombin cleavage. The supernatant, purified proteins, and elution waste fractions were analyzed by SDS-PAGE with a control sample from transformant of the empty pET-28b (+) vector (Fig. 1). His-tag purification led to highly purified protein, and thrombin cleavage further purified the protein and led to shorter peptide without the N-terminal fusion residues as shown by its decreased molecular mass.

### Substrate preference of BLC23O from strain L1

Substrate preference of BLC23O was investigated by monitoring the UV–vis spectral peaks over time. Appearance of absorbance peak at a specific λmax on the UV–vis spectrum and increased absorbance intensity over time indicated a continuous activity of BLC23O on the substrate. Among the tested substrates, BLC23O was active on a few substrates as listed in Table 1. The UV–visible spectra and maximum wavelengths of the expected products from six of the active substrates are shown in Fig. SI3 (A-F) and their corresponding no-enzyme controls are shown in [Fig. SI3 (A_1_-F_1_)]. A significant increase in the product peak (λmax 390 nm) as compared to the control (Fig. SI3A_1_) from 3-ethylcatechol was observed between 0 and 30 min of reaction times (Fig. SI3A) consistent with extradiol cleavage of 3-ethylcatechol by C23O enzymes as reported by literature. When cleavage products were detectable, the specific λmax for other tested compounds all agreed with literature reported wavelengths^45–47^ confirming BLC23O enzymatic activities. The other tested substrates did not show visible peak or noticeable change in spectra [Fig. SI4 (A-F)], as compared to no-enzyme control [Fig. SI4 (A_1_-F_1_)]. Different from the other 5 substrates that showed expected product peaks, 4-chlorocatechol did show spectral changes with enzyme addition, however, no specific peak corresponding to the expected cleavage product at 379 nm^43^ was observed.

**Table 1:** Comparison of BLC23O substrate preference with literature reported C23Os.

Substrate	BLC23O*	C23O-K11	C23O1-Hb	C23O2-Hb	C23O-P5	C23O-Sm	C23O-P47	C23O-Pp	C23O-Ps
Catechol	100	100	100	100	100	100	100	100	100
3-methylcatechol	4000 ± 6	90	49	239	13	10	33	10	40
4-methylcatechol	170 ± 2	67	87	280	106	82	62	41	70
4-chlorocatechol	120 ± 2	71	50	226	203	0	71	62	–
2,3-dihydroxybiphenyl	3100 ± 50	0	–	–	–	–	–	–	–
3-fluorocatechol	0	–	–	–	–	–	–	–	–
Protocatechuic acid	0	–	–	–	–	–	–	–	–
2,5-dihydroxybenzoic acid	0	–	–	–	–	–	–	–	–
1,2-dihydroxynapthalene	0	–	0	0	–	–	–	–	–
3,4-dimethylcatechol	–	–	–	–	–	–	–	–	22
3-isopropylcatechol	3200 ± 43	–	–	–	–	–	–	–	–
3-ethylcatechol	6400 ± 290	–	–	–	–	–	–	–	–

*Kinetic assays were performed with 1 mM of corresponding substrate in 0.1 M Tris–HCl (pH 7.4) buffer, 60 µg/mL BLC23O and 0.1 mM MnCl_2_·4H_2_O. Absorbance was measured at the wavelength of the corresponding expected cleavage product. The molar extinction coefficient and wavelength employed for each cleavage product listed in “section Substrate preference of BLC23O from strain L1”. All numbers are expressed as percentage specific activities with catechol set as 100%. The specific activity of BLC23O reaction with catechol was determined as 0.0082 µmol·mg-E^−1^ ·min^−1^ (ε = 33,400 M^−1^·cm^−1^, 375 nm and pH 7.6). C23O-K11 is from *Thauera* sp. K11^54^; C23O1-Hb and C23O2-Hb are from a hallophilic bacterial consortium^47^; C23O-P5 is from *Planococcus* sp.S542^41^; C23O-Sm is from *Stenotrophomonas maltophilia* KB243^53^; C23O-P47 is from Pseudomonas sp. S-4748^80^; C23O-Pp is from *Pseudomonas putida* mt-2^81^; C23O-Ps is cell extract from *o*-xylene grown *Pseudomonas stutzeri* strain^82^. 0 means no activity detected; – means not tested.

Based on absorbance readings at specific product wavelength, the relative BLC23O activities towards 12 tested aromatic compounds were shown in Table 1. BLC23O showed activity against catechol, 3-ethylcatechol, 3-methylcatechol, 3-isopropylcatechol, 4-methylcatechol, 4-chlorocatechol, and 2, 3-dihydroxy biphenyl. The maximum activity of BLC23O was observed against 3-ethylcatechol, reasonable activity was observed against 3-methylcatechol, 3-isopropylcatechol, 2, 3-dihydroxybiphenyl, 4-methylcatechol. A modest BLC23O activity was shown for catechol and 4-chlorocatechol. These results revealed that 3-ethylcatechol was the best substrate for the BLC23O. Compared to other literature reported C23Os, BLC23O showed the best relative activity for 3-methylcatechol over catechol (40-fold, 3-methylcatechol/catechol). Interestingly, even higher relative activity (64-fold) was observed for 3-ethylcatechol. In addition, 3-isopropylcatechol showed 32-fold relative activities over catechol. For 4-methyl catechol, less than 2-fold relative activity was observed. These results suggested that BLC23O can cleave the catechol ring when substitutions occur at C3 more efficiently than C4 substitutes. Interestingly, 31-fold activity towards 2,3-dihydroxybiphenyl over catechol was also observed suggesting that BLC23O can cleave aromatic compounds with two 6-carbon ring structures. Examination of 3-dihydroxybiphenyl chemical structure showed that it has a phenol ring substitution adjacent to the two HO-groups on the other C6 ring, similar to the catechol C3 substitution, further confirming that BLC23O is more efficient against C3 substitution substrates. However, a fused 6-carbon double ring structure like 1,2-Dihydroxynaphthalene could not be cleaved even though it does have two –OH groups like catechol. In addition, no activities were observed for catechol C3-substitutions by –OH (pyrogallol) and –F (3-fluorocatechol) suggesting the preference for substrates with non-ionic substitutions. Also, the results showed a possible degradation of halocatechols by BLC23O at slightly higher activity against 4-chlorocatechol as compared to catechol^50,51^. However, since the specific cleavage product peak was not as obvious as other substrates, the detailed activity and cleavage product of 4-chlorocatechol need to be further confirmed.

### Effect of temperature and pH on BLC23O activity

**Figure 2:**
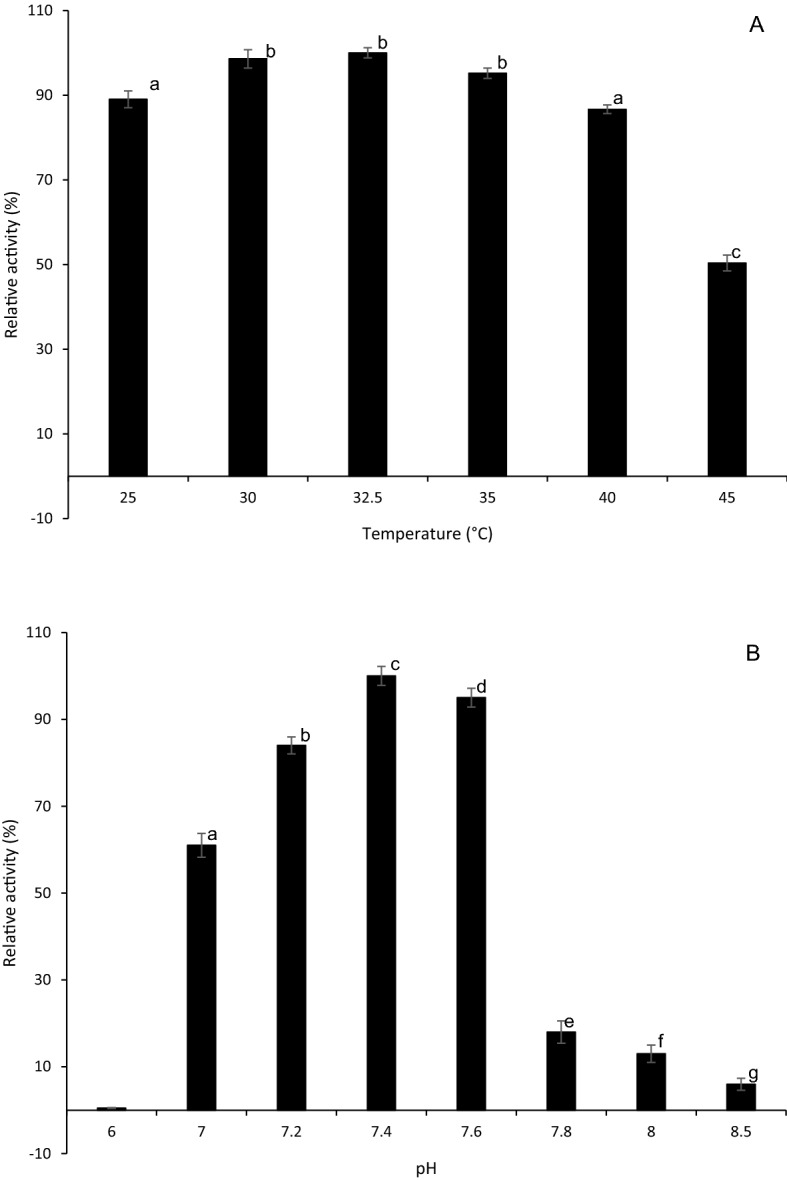
Effect of temperature and pH on BLC23O activity. (A) Temperature; (B) pH. The error bars represent the standard deviation from triplicate experiments. Means of the bar with different letters significantly differ (p < 0.05).

**Figure 3:**
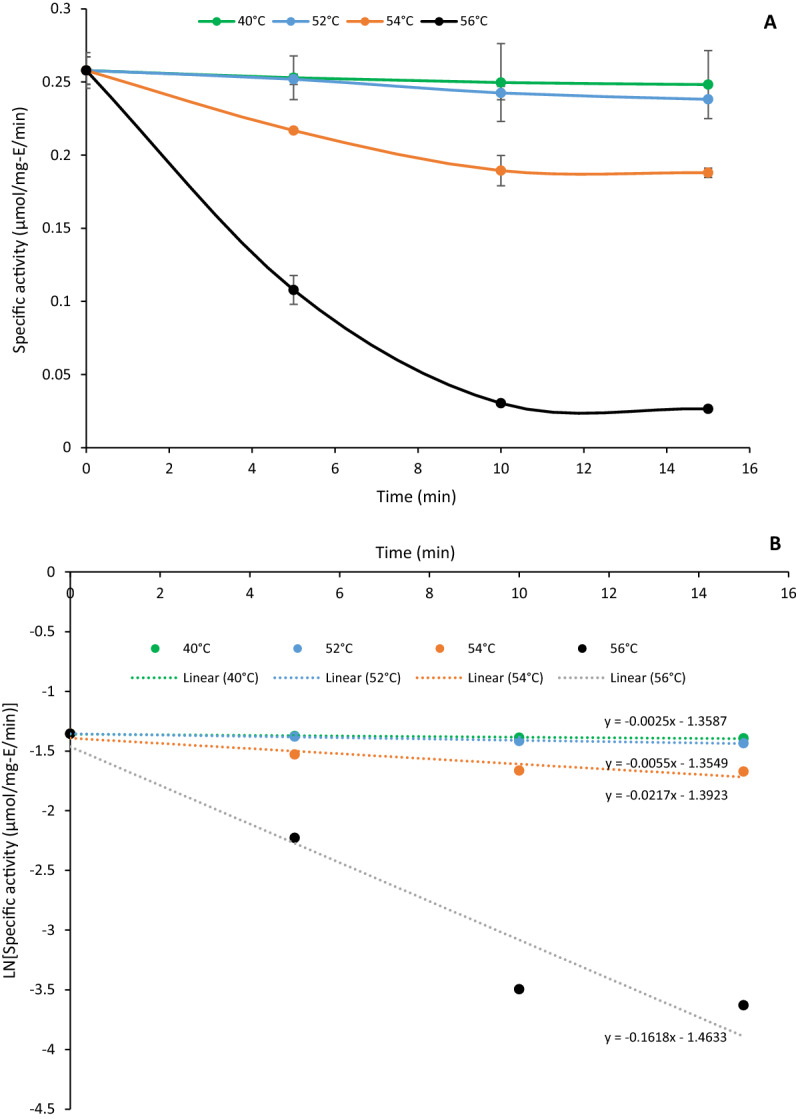
Determination of thermostability parameters. (A) BLC23O thermal stability; and (B) Linear regression curve. To determine* k*_d_ for the enzyme half-life estimation by incubating the enzyme in triplicate using 1 mM 3-methylcatechol in 0.1 mM MnCl_2_·4H_2_O in 0.1 M Tris–HCl buffer (pH 7.4). The enzyme was pre-incubated at 40, 52, 54, and 56 °C for different time lengths.

The impact of reaction temperature on enzyme activity was examined at the temperature range of 25–45 °C using 3-methylcatechol, one of the active substrates that has been commonly used for other enzyme activity assays. The enzyme showed maximal activity at 32.5 °C that decreased with increased temperature (Fig. 2A). There was no significant difference (*p* > 0.05) between the relative activity of BLC23O at 30 °C, 32.5 °C, and 35 °C, but the specific activity of BLC23O at 32.5 °C was slightly higher than the other two temperatures. At 45 °C, the enzyme activity was around 60% of the specific activity at the optimal temperature. BLC23O was active at pH ranging from 7 to 8.5 with its optimal specific activity at pH 7.4 (Fig. 2B). The enzyme was not active at very low pH (3–6) and very high pH (9–11). There was no significant difference (p > 0.05) in the BLC23O activity between pH 7.4 and 7.6. The results revealed BLC23O was active under neutral and weak alkaline conditions. This property is suitable for its practical application without needs of much chemicals to adjust pH from compound water solution. The extinction coefficient to calculate the specific activity of the product cleavage of BLC23O reaction with catechol was obtained at pH7.6 since there were no significant difference in BLC23O activities between pH7.4 and 7.6 (Table 1). To determine enzyme thermal stability (half-life), linear regression curves and the experimental data were plotted in Fig. 3. The plot ordinates were normalized, represented by the specific activity of the BLC23O thermal stability at 40, 52, 54, and 56 °C (Fig. 3A). The model (Fig. 3B) predicted the enzyme activity quite well according to the changes in temperature. The *k*_d_ value of BLC23O was estimated as 0.003, 0.006, 0.022, and 0.162 min^−1^ at 40, 52, 54, and 56 °C, respectively. The obtained values of *k*_d_ were substituted into Eq. 2 to determine BLC23O half-life (t_1/2_) and the t_1/2_ of BLC23O was determined as 231, 116, 32, and 4 min at 40, 52, 54, and 56 °C, respectively. The incubation temperature of 40 °C had the lowest degradation rate constant *k*_d_, indicating that BLC23O deactivated more slowly at this temperature. The half-life of BLC23O at 40 °C was 2-fold relative to 52 °C, 7-fold relative to 54 °C and 58-fold relative to 56 °C. Incubation at 32.5 and 35 °C for 3 h did not show any activity decrease (data not shown) suggesting that BL230 is very stable at ~ optimum temperatures.

### Effect of metal ions on BLC23O activity

The effect of six different metal ions on BLC23O activity was investigated using 3-methylcatechol as the substrate (Table 2). Most of the metal ions examined in this study increased the enzyme activity except Mg^2+^ and K^+^ that did not show obvious impact on BLC23O activity.

**Table 2:** Comparison of metal ions effects on BLC23O activity for 3-methylcatechol (without metal ions expressed as 100% relative activity, values represent the average of triplicate experiments).

Metal ions	Relative activity of BLC23O at two metal concentrations	
	0.1 mM	1.0 mM
Control	100	100
Cu^2+^ CuSO4	400 ± 10	1000 ± 15
Fe^2+^ FeSO_4_·7H_2_O	770 ± 16	2200 ± 120
Fe^2+^ FeSO4·7H2O 5 mM Sodium ascorbate	930 ± 14	2000 ± 35
Fe^3+^ FeCl_3_	570 ± 28	4000 ± 32
Mg^2+^ MgCl_2_.6H_2_O	98 ± 3	120 ± 9
K^+^ KCl	100 ± 3	110 ± 8
Mn^2+^ MnCl_2_.4H_2_O	17,000 ± 480	23,000 ± 570

The minor impacts of K^+^ and enhanced activity by Fe^2+^, Fe^3+^, and Cu^2+^ are consistent with reported results by Guo et al.^47^. While Guo et al.^47^ reported minor impacts by Mn^2+^, Silva et al.^52^ reported 2.3 times enhancement. The discrepancies may be indeed due to different enzyme characteristics. However, different experimental processes may also lead to misinterpretation. For example, different buffers and concentrations of metals used by different groups may both affect the results. Published literature used Tris buffer^52^ or phosphate buffer^28,47,53^ and metal concentrations ranged from 1–3 μM^53^, 0.01 mM^28^ to 5 mM^47,54^. We used the intermediate concentrations of 0.1 and 1 mM for each metal and found some inconsistencies. For example, the impacts of three metals (Fe^2+^, Cu^2+^, and Fe^3+^) increased significantly at higher concentration while Mg^2+^, K^+^, and Mn^2+^ only led to slight increase at higher concentration, and the increase was not in a dose-dependent manner. Non-dose dependent impacts with increased metal concentrations were also shown by Wojcieszyńska et al.^53^. It is possible that some metals may precipitate at higher concentration during the assay periods. Therefore, proper soluble concentration for each metal may need to be determined.

In this study, we tried to use lower metal concentration and BLC23O showed the largest activity in the presence of Mn^2+^ that contradicts to Guo et al.^47^ who showed that Mn^2+^ did not affect the two C23Os activities. To be sure of our results, the experiments were repeated several times independently by two associates. We confirmed that at 1 mM concentration, Mn^2+^ increased the BLC23O enzyme activity by 230 times as compared to the reaction without metal. This suggested that the as-purified enzyme may contain very little metal ion in its active site. Indeed, when metal contents were analyzed (20 different metal ions), the as-purified BLC23O contained very little metal mostly below the Method reporting limit (MRL). Both Fe^2+^ and Mn^2+^ were below MRL (Table SI1).

So far, intradiol dioxygenases have been found to contain Fe (III) at the active centers, and most extradiol dioxygenases have been found to contain Fe (II) with a few exceptions of Mn (II)-dependent enzymes^55–58^. We have shown that BLC23O activity is much more enhanced by Mn^2+^ than by Fe^2+^. To make sure the impact of Fe^2+^ is not affected by oxidative inactivation, reducing agent sodium ascorbate was included in another set of activity assays (Table 2). Compared to the same assay without sodium ascorbate, the impact of Fe^2+^ at 0.1 mM was slightly increased (20%) but no difference at 1 mM concentration suggesting that reducing reagent may be needed for lower Fe^2+^concentration. Regardless, the impact of Mn^2+^is 18-fold that of Fe^2+^ at 0.1 mM concentration confirming that BLC23O is a Mn^2+^-dependent extradiol dioxygenase.

The first oxygen-activating manganese enzyme, 3,4-Dihydroxyphenylacetate 2,3-dioxygenase (Bacillus Mn C23O, Accession WP_019716166) , was reported by Que et al.^57^. The Mn(II)-dependent 3,4-dihydroxyphenylacetate (3,4-DHPA) 2,3 dioxygenase was characterized from *Arthrobacter globiformis* CM-2 by Boldt et al.^55^. Similarly, Whiting et al.^58^ reported a manganese-dependent 3, 4-dihydroxyphenylacetate 2, 3-dioxygenase (MndD) from *Arthrobacter globiformis* strain CM-2. Different from Fe(II)-dependent dixoygenases, all three reported enzymes are not inactivated by H_2_O_2_ but inhibited by ferrous iron. Differently, we found BLC23O activity is not inhibited by Fe(II). A thermostable Mn(II)-dependent 2,3-dihydroxybiphenyl-1,2-dioxygenase from the thermophilic biphenyl and naphthalene degrader, *Bacillus* sp. BphC-JF8 was reported to have substrate preference in the order of 2,3-dihydroxybiphenyl > 3-methylcatechol > catechol > 4 methylcatechol > 4-chlorocatechol^56^. Even though this order is different from BLC23O, it does show similarity in that both enzymes have activity against the four substrates and both enzymes cleaved the bicyclic substrate more efficiently than the chloro-modified catechol.

**Figure 4:**
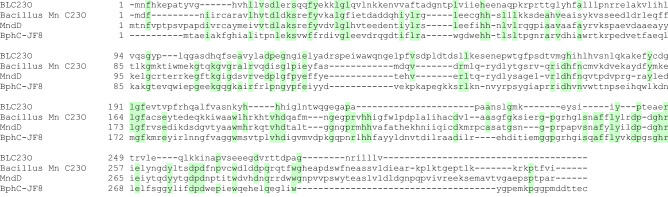
Sequence comparison of BLC23O with other Mn-dependent dioxygenases. Comparison was performed by Multi-Way, exhaustive pairwise alignment of all sequences and progressive assembly of alignments using Neighbor-Joining phylogeny, scoring matrix Blosum 62, with Clone Manager 9 (Scientific & Educational Software).

The amino acid sequence of BLC23O was compared with the other three characterized Mn-dependent dioxygenases and very little similarity was shown (Fig. 4). Multiple sequence comparison (Multi-way protein alignment) of the other three enzymes showed that Bacillus Mn C23O and MndD had 50% and 48% sequence matches whereas BphC-JF8 showed only 33% match. Pairwise sequence comparison (Emboss Needle) showed that BLC23O has identity (similarity, gaps) of 11% (18%, 70%); 16% (25%, 47%); and 19% (32%, 30%) with Bacillus Mn C23O; MndD, and BphC-JF8, respectively. Therefore, BLC23O is a unique Mn-dependent dioxygenase so far characterized.

The detailed mechanism of active site metal preference is not clear. Iron and manganese -dependent enzymes share high sequence homology and similar structures, especially near the active site as shown by X-ray structures of two isofunctional extradiol dioxygenases, Mn(II)-dependent MndD from *Arthrobacter globiformis* and Fe(II)-dependent HPCD from *Brevibacterium fuscum*^59^. Given the differences of substrate specificities and the unique metal impact on enzyme activity compared with other reported Mn(II) dependent dioxygenases, BLC23O may also have unique structural features and functionality. Detailed comparison of BLC23O structure with other structurally characterized dioxygenases may give new insight into the roles of the peripheral and active-site residues and the different mechanisms of specific ring cleavage by the extradiol dioxygenase family. Substrate specificity is related to enzyme structure whereas metal preference is particularly related to the catalytic center structure^60^. We are currently working on resolving its crystal structure which may shed light on the understanding of its catalytic mechanisms that will also help developing its applications in lignin valorization and phenolic compound biodegradation.

### Phylogenetic analyses

Phylogenetic analyses with other extradiol dioxygenases were performed. When “C23O” was used to search the NCBI protein database, totally 954 items were identified. Total 25 sequences representing most of the full-length, non-redundant lists were selected and compared with BLC23O by phylogenetic analyses (Fig. SI5). BLC23O is only related to one sequence, accession MK240185.1 from *Bacillus licheniformis*, with a 42% sequence identity, 57% similarity, and 6% gaps (pairwise sequence alignment with EMBOSS Needle, Matrix EBLOSUM62). The rest of the sequence with a sequence identity of < 20%. Compared with the characterized C23O, YfiE (CatE), from *Bacillus subtilis* 168^61^, BLC23O shows 41% amino acid sequence identity (59% similarity). Further comparison with 29 *Bacillus* C23O sequences did not group BLC23O to any specific sequence as illustrated in the supporting information (Fig. SI6A). The closest sequence (accession SAE32001) showed a 51% sequence identity but overall sequence similarity is much different (Fig SI6B).

**Figure 5:**
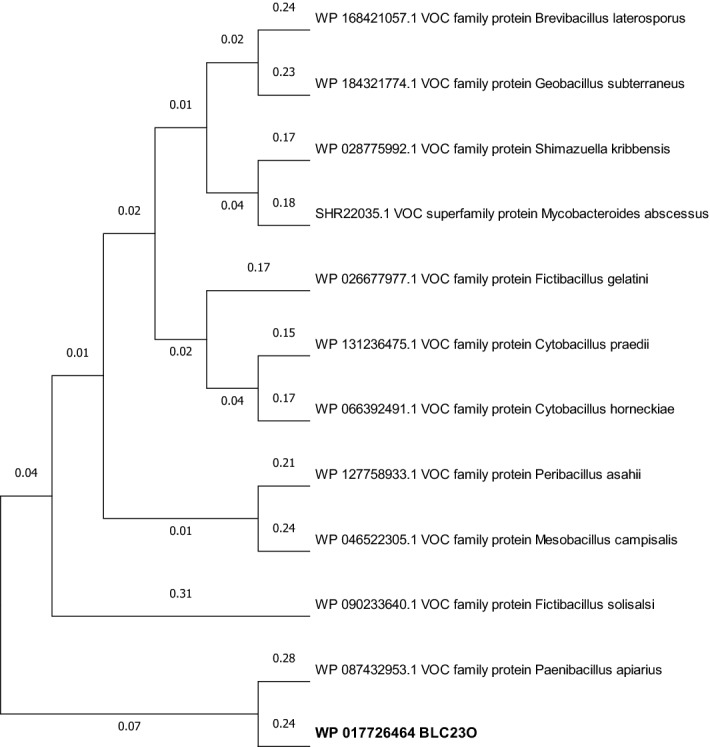
Phylogenetic analyses of BLC23O with other VOC family proteins. The evolutionary history was inferred using the Neighbor-Joining method^77^. The optimal tree is shown. The evolutionary distances were computed using the Poisson correction method^78^ and are in the units of the number of amino acid substitutions per site. This analysis involved 12 amino acid sequences. All ambiguous positions were removed for each sequence pair (pairwise deletion option). There were a total of 359 positions in the final dataset. Evolutionary analyses were conducted in MEGA-X^79^.

Blasting search using BL23O amino acid sequence identified a list of mostly unidentified VOC family protein with protein sequence identities of > 50%. Eleven non-redundant sequences from different strains were selected for comparison and BLC23O was grouped with an uncharacterized protein (WP_087432953) from *Paenibacillus apiarius* with 64% amino acid sequence identity (Fig. 5). These analyses suggested that BLC23O forms a new subgroup with the uncharacterized sequence WP_087432953.

### Reaction kinetics model and rate constants

**Figure 6:**
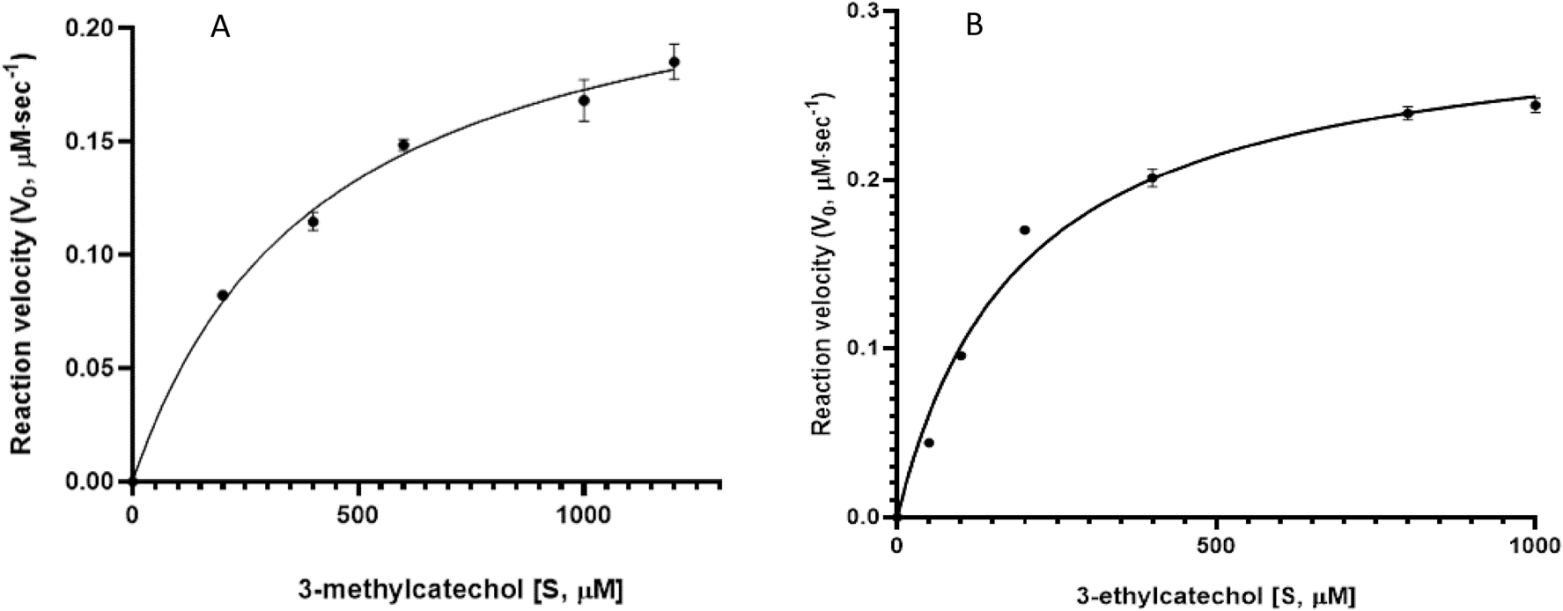
Michaelis–Menten plot for substrate cleavage by BLC23O. (**A**) 3-methylcatechol; (**B**) 3-ethylcatechol. A substrate range of 200 to 1200 µM was chosen for the kinetic assay. The parameter results were obtained from triplicate experiments.

Figure 6 shows the Michaelis–Menten kinetics plot of BLC23O with 3-methylcatechol (Fig. 6A) or 3-ethylcatechol (Fig. 6B) as substrate. The *K*_m_, *k*_cat_, *k*_cat_/*K*_m_ of BLC23O towards 3-methylcatechol and 3-ethylcatechol were determined as 418 µM, 0.2 s^−1^, < 0.01 s^−1^ µM^−1^ and 193 µM, 0.5 s^−1^, < 0.01 s^−1^ µM^−1^, respectively (Table 3A). The estimated *K*_m_ of BLC23O towards 3-methylcatechol were more than 2-fold higher than 3-ethyl catechol, but the catalytic efficiency (*k*_cat_/*K*_m_) of BLC23O towards both substrates was five times different (Table 3A). The comparison of kinetic constants of various EDOs with BLC23O using 3-methyl catechol as substrate is shown in Table 3B. Both the *K*_m_ and the catalytic efficiency value (*k*_cat_/*K*_m_) of BLC23O were the lowest among all listed enzymes.

**Table 3:** BLC23O catalytic properties towards 3-methyl catechol and 3-ethyl catechol (A); and comparison of the kinetic constants of various extradiol dioxygenases with BLC23O towards 3-methylcatechol (B).

Substrate	BLC23O from *Bacillus ligniniphilus* sp Strain L1				
	K_m_ (µM)	K_cat_ (s^−1^)	K_cat_/K_m_ (M^−1^ s^−1^)		
(A)					
3-methyl Catechol	418 ± 25	0.20 ± 0.03	4.8 × 10^2^		
3-ethyl Catechol	193 ± 17	0.47 ± 0.09	2.4 × 10^3^		
(B)					

Enzyme*	Organism	K_m_ (μM)	k_cat_ (s^−1^)	k_cat_/K_m_ (M^−1^ s^−1^)	References
C23O_WT_	*Pseudomonas putida* KT2440	1.7	476	2.8 × 10^8^	Cerdan et al.^83^
C23O _XylE_	*Pseudomonas putida* KT2440	1.8	490	2.7 × 10^8^	Cerdan et al.^84^
C23O _Tyr218_	Pseudomonas sp. 1YB2	1.5	17	1.1 × 10^7^	Junca et al.^85^
C23O _His218_	Pseudomonas sp. 1YB2	3.2	25	8.0 × 10^6^	Junca et al.^85^
C23O _XylE_	*Pseudomonas putida* mt-2	1.6	221	1.4 × 10^8^	Kobayashi et al.^86^
C23O_HA10_	*Pseudomonas sp.* HA10	35	200	5.7 × 10^6^	Hassan and Aly^34^
Tcu3516	*Thermomonospora curvata* DSM43183	20	65	3.3 × 10^6^	Zhang et al.^87^
C23O_Mpc_	*Pseudomonas putida mt-2*	1.6	221	1.4 × 10^8^	Ishida et al.^88^
BLC23O	*Bacillus ligniniphilus* L1	418 ± 25	0.2 ± 0.03	4.8 × 10^2^	This study

*Using 3-methylcatachol as substrate, BLC23O has a K_m_ value of 418 micromole, while the K_cat_ and K_cat_/K_m_ values are the lowest among reported.

Due to its unique sequence based on homology analyses and metal dependency, it is possible that the best substrate had not been tested even though a reasonable list of commonly used substrates had been tried. In addition, the monomeric status of BLC23O (this paper) may be one reason for lower enzyme activity as shown by low substrate affinity (higher K_m_). It is possible that polymeric forms allow synergistic interactions between subunits to enhance both substrate binding and product cleavage rate. Interestingly, compared to BLC23O, the other reported monomeric EDO (2,2',3-THB dioxygenase) has 13 times higher K_m_ towards 3-methylcatechol (5.3 mM)^62^. However, 2,2',3-THB dioxygenase also has much lower K_m_ towards its preferred substrates (8.5, and 11 μM for 2,3-DHB and 2,2',3-THB respectively). This observation may support a hypothesis that the correct substrate for BLC23O is yet to be identified.

Regardless, six of the tested substrates showed unique cleavage product formation and seven showed obvious absorption increase suggesting that indeed BLC23O have activities against these substrates.

### Oligomeric state and potential involvement of BLC23O in aromatic compound catabolism

Native EDOs have been reported to be homohexamers^63,64^ and octamers^65–67^. The first 3D structure of C23O from *Psedomonas putida* mt-2 shows a homotetramer structure^68,69^. Activity analyses of hybrid C23Os show that only tetrameric forms but not the monomeric form have activity suggesting that intersubunit interaction to ensure the tetramer formation is critical for C23O activity^69^.

**Figure 7:**
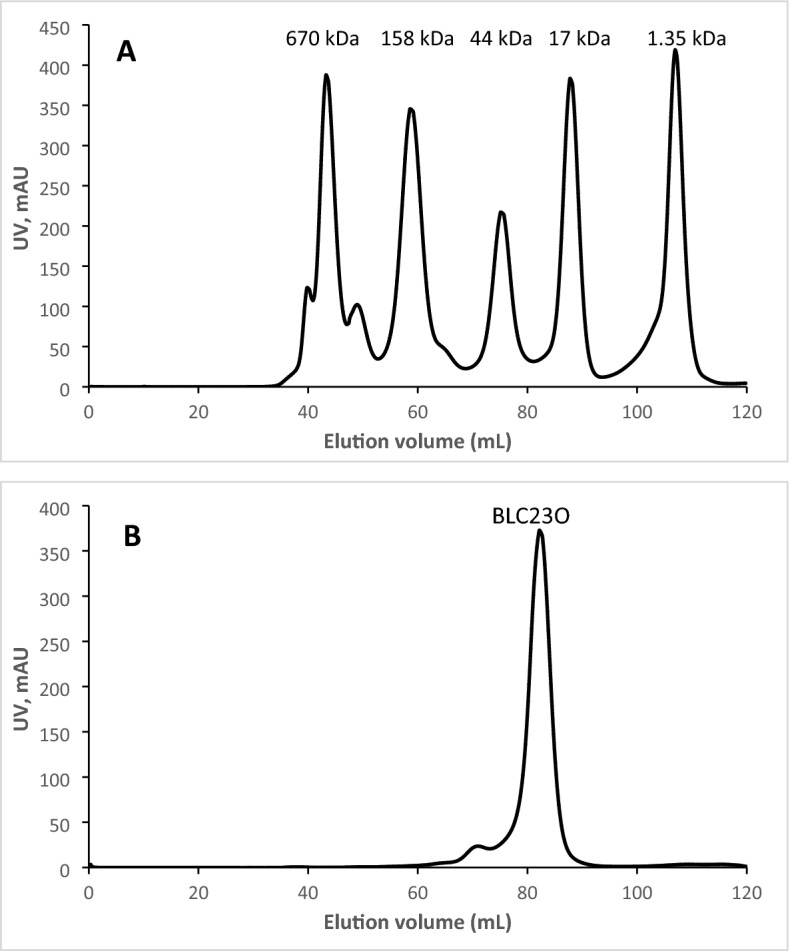
HPLC chromatogram of BLC23O. (**A**) Gel filtration protein standard. Thyroglobulin (bovine) 670 kDa; γ-globulin (bovine) 158 kDa; Ovalbumin(chicken) 44 kDa; Myoglobin (horse)17 kDa; VitaminB12, 1.35 kDa. (**B**) Purified BLC23O.

Many enzymes function as oligomers but not in monomeric forms with mechanisms not fully understood. The possible mechanisms for the oligomerization-dependent activity of enzymes are protein stabilization, active sites formation, and conformational changes upon oligomerization^70^. To confirm if BLC23O also forms oligomers like reported enzymes, HPLC gel filtration analyses were performed using native BLC23O protein and compared with native MW markers. Unexpectedly, only one major peak was eluted between the 17 and 44 kDa markers with an estimated MW of ~ 32 kDa indicating native BLC23O is under monomeric state [Fig. 7]. This is unusual as so far, only one EDO has been reported to be monomeric^62^.

**Figure 8:**
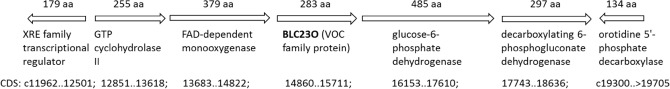
ORFs surrounding BLC23O coding gene.

The low catalytic activity of BL23O (shown by kinetic assays) raised a question whether this enzyme is practically involved in cellular metabolism of aromatic compounds. Genomic analysis of surrounding genes may give some indication of its functionality. As reported by Bugg and Winfield^39^, in bacteria, catechols are commonly produced by ortho-hydroxylation of phenols, carried out by FAD-dependent monooxygenases. After ring cleavage by dioxygenases, completion of the catechol meta-cleavage pathway includes dehydrogenase and decarboxylase as reported in *Pseudomonas putida* mt-2. Blast searching of the BLC23O encoding gene (BLT_RS0103695) and its up- and down-stream orfs identified enzymes involved in the reported pathway (Fig. 8). As reported in other bacteria, FAD-dependent monooxygenase catalyzes the formation of catechols from phenolic compounds that are produced after lignin degradation.

However, the glucose-6-phosphate dehydrogenase and decarboxylating 6-phosphogluconate dehydrogenase genes, located near the BLC23O gene, are pentose phosphate pathway enzyme genes. They may not be involved in the catabolism of ring cleavage products of catechols. It is therefore difficult to predict what aromatic compounds BLC23O gene is involved in catabolizing.

As reported by Zhu et al.^25^, strain L1 has 3 C23O coding sequences and 3 pathways for lignin degradation, it is possible that BLC23O is needed for the bacterial growth on some unidentified substrate from lignin degradation pathway different from the other two C23Os. Alternatively, BLC23O may play a unique supporting role while one or two of the other enzymes may be essential for strain L1 growth. For example, among the 8 EDO genes in *Rhodococcus* sp. K37, only *BphC8* is essential for its growth on 2,3-Dihydroxylbiphenyl (biphenyl)^71^. Similarly, based on gene disruption, BphC2 is essential for *Rhodococcus* sp. R04 growth on biphenyl while BphC1 plays an assistant role. Consistently, BphC2 is 43-fold active (*K*_cat_/*K*_m_ value) compared to BphC1^63^.

These results suggested that BLC23O not only has unique sequence, metal preference, and activities against different substrates but also unique monomeric structure, compared with reported EDOs. Its monomeric native structure allows good potential to be conveniently engineered for practical applications in a wide range of substrate cleavage and product formation. Such kinds of oxidative enzymes can be used in an integrated process for lignin valorization wherein a “biological funneling” step for lignin depolymerization is followed by enzymatic upgrading of aromatic compounds^38,72–76^. In addition, aromatic compound cleavage enzymes can also be applied for treatment of waste water contaminated with phenolic compounds.

## Conclusions

The first dioxygenase from Strain L1 was successfully expressed, purified, and characterized with T_opt_ of 32.5 °C and pH_opt_ of 7.4. Compared to other C23Os, BLC23O showed some unique features such as (1) instead of Fe^2+^-dependent, BLC230 is Mn^2+^- dependent; (2) unique and wide substrate activities with the highest (40-fold) 3-methylcatechol/catechol relative activity compared to other reported enzymes and a 31-fold cleavage activity of a bicyclic phenolic ring compound over catechol; (3) unique aa sequence as compared to other characterized C23Os and Mn-dependent dioxygenases and may form a new subgroup with an uncharacterized VOC family protein from *Paenibacillus apiaries;* (4) unique monomeric active form different from reported EDOs and with significant thermostability (long half-life) that may allow this enzyme to be easily engineered to improve activity against a wide range of substrates for practical product formation.

## Supplementary Information

Supplementary Information.
