# Supplementary material for: A novel Bacillus ligniniphilus catechol 2,3-dioxygenase shows unique substrate preference and metal requirement
Source: Sci Rep. 2021 Dec 14;11:23982. doi: 10.1038/s41598-021-03144-8 (PMC8671467; doi:10.1038/s41598-021-03144-8)
Supplement: Supplementary file 1 — Supplementary Information. [file 41598_2021_3144_MOESM1_ESM.docx]

**Supplementary information**

ATGAACTTCCACAAAGAACCGGCTACCTACGTTGGTCACGTTCACCTGCTGGTTTCCGACCTGGAACGTTCTCAGCAGTTCTATGAGAAAAAACTGGGTCTGCAGGTTCTGAACAAAAAAGAAAACGTTGTTGCATTCACCGCTGACGGTAACACCCCGCTGGTTATCATCGAACACGAAGAAAACGCTCAGCCGAAACGTCCGCGTACCACCGGTCTGTACCACTTCGCTCTGCTGCTGCCGAACCGTCGTGAACTGGCGAAAGTACTGATCCACCTGGTTCAGTCTGGTTACCCGCTGCAGGGTGCGTCTGACCACCAGTTCTCTGAAGCGGTTTACCTGGCTGACCCGGAAGGCAACGGTATCGAACTGTACGCGGACCGTAGCCCGGAAATCTGGGCATGGCAGAACGGTGAACTGCCGTTCGTTTCTGACCCGCTGGACACCGACTCCCTGCTGAAAGAATCTGAAAACGAACCGTGGACCGGTTTCCCGTCTGACACCGTAATGGGTCACATCCACCTGCACGTTTCTAACCTGCAGAAAGCTAAAGAATTCTACTGCGACGGCCTGGGCTTCGAAGTTACCGTTCCGTTCCGTCACCAGGCGCTGTTCGTTGCTTCTAACAAATACCACCACCACATCGGTCTGAACACCTGGCAGGGTGAAGGTGCACCGGCACCGGCGGCTAACTCTCTGGGTATGAAAGAATACTCCATCATCTACCCGACCGAAGCTGAACGTACCCGTGTTCTGGAACAGCTGAAGAAAATCAACGCACCGGTTTCTGAAGAAGAAGGTGACGTTCGTACCACCGACCCGGCTGGTAACCGTATCCTGCTGCTGGTTTAA

**Fig. SI1.** BLC23O nucleotide sequence

cs cp s p s p s p s p s p

37◦C

16◦C

1 0.01 1 0.1 0.01


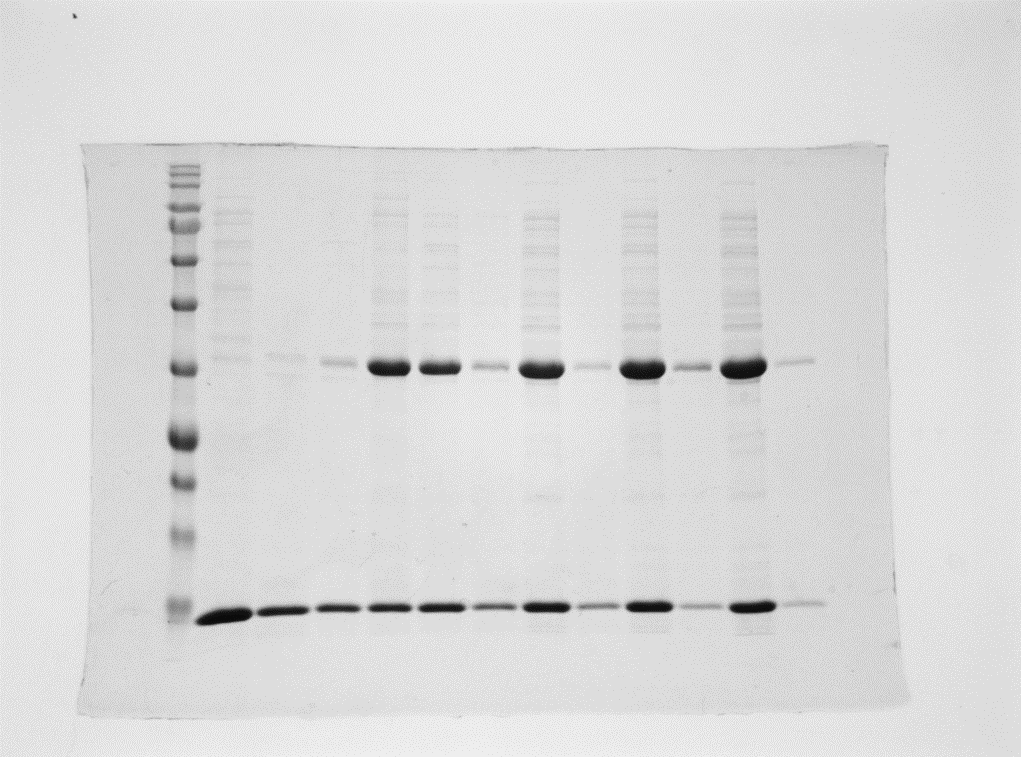


32

46

58

25

22

17

11

kDa

M

**Fig. SI2.** Optimization of soluble protein expression for BLC23O. Prior to lysis, the O.D.s of the cultures were brought to ~0.4 and equal volumes were used to estimate an equal amount of cells. The lysates (“s”) and pellets (“p”) were suspended in an equal volume of buffer and equal volumes of each cell fraction were loaded on a 12% SDS-PAGE gel to determine the solubility of overexpressed protein. Culturing temperatures were listed above numerical IPTG concentrations, M is the molecular mass marker with size labeled beside bands, and “c” is a control (cells transformed with the empty vector).


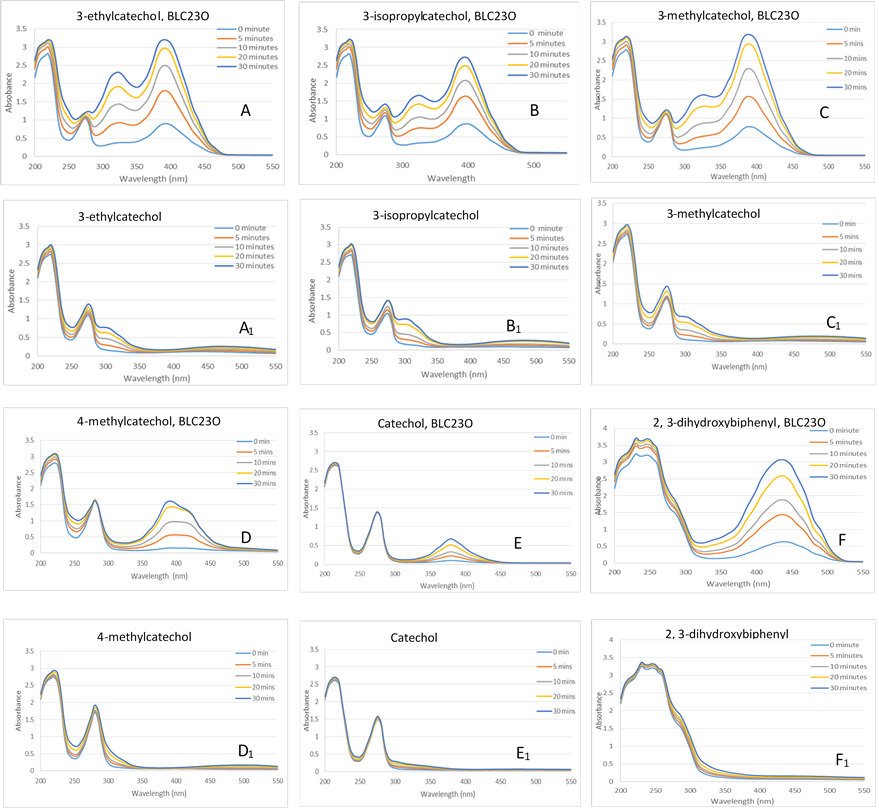


**Fig. SI3 (A-F).** UV-visible spectra and maximum wavelengths of the expected BLC23O cleavage products of active catecholic substrates. Spectra with no subscripted letter label represent substrates in buffer solution with enzyme; spectra with subscripted letter label represent control reactions - substrates in buffer solution without enzyme. The spectra were obtained in 0.1M Tris-HCl (pH 7.4) buffer at 32.5°C with the addition of 0.1mM MnCl_2_·4H_2_O and 90 µg/mL enzyme using 1mM each of the aromatic compounds as substrates. The UV-vis (200-550 nm) spectra of the enzyme reaction mixtures and the control reactions were captured at 2 or 5 nm steps and a series of time points from 0 to 30 minutes. Each graph curve represents a different reaction time and is the mean of triplicate measurements.


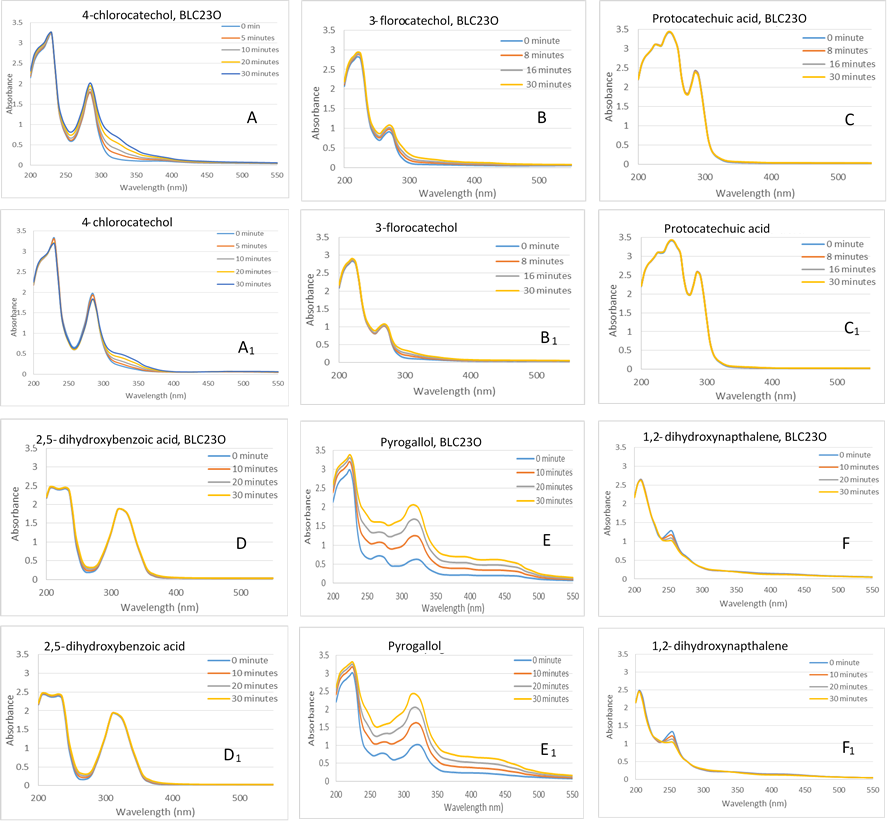


**Fig. SI4 (A-F).** UV-visible spectra of inactive catecholic substrates. Spectra with no subscripted letter label represent substrates in buffer solution with enzyme; spectra with subscripted letter label represent control reactions - substrates in buffer solution without enzyme. The spectra were obtained in 0.1M Tris-HCl (pH 7.4) buffer at 32.5°C with the addition of 0.1mM MnCl_2_·4H_2_O and 90 µg/mL enzyme using 1mM each of the aromatic compounds as substrates. The UV-vis (200-550 nm) spectra of the enzyme reaction mixtures and the control reactions were captured at 2 or 5 nm steps and a series of time points from 0 to 30 minutes. Each graph curve represents a different reaction time and is the mean of triplicate measurements.

**
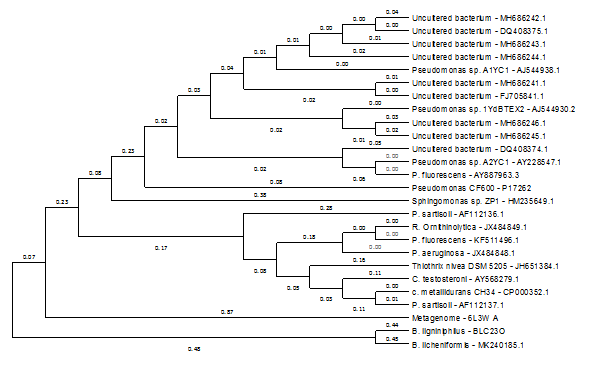
**

**Fig. SI5.** Phylogenetic analysis of BLC23O with other extradiol dioxygenases. The evolutionary history was inferred using the Neighbor-Joining method^1^. The optimal tree is shown. The evolutionary distances were computed using the Poisson correction method^2^ and are in the units of the number of amino acid substitutions per site. This analysis involved 26 amino acid sequences. All ambiguous positions were removed for each sequence pair (pairwise deletion option). There were a total of 359 positions in the final dataset. Evolutionary analyses were conducted in MEGA X ^3^

**
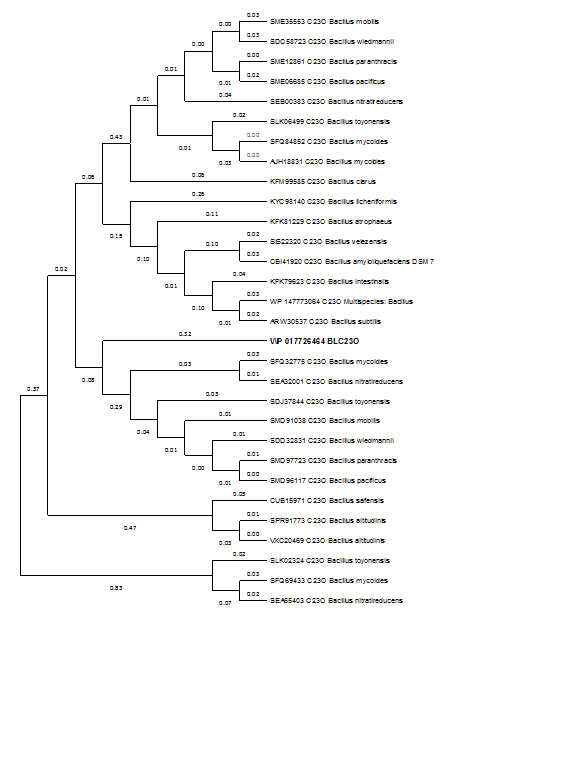
**

**A**

**B**

Areas of significant similarity (window length 5 bases)

BLC23O

SAE32001

SDJ37844

SMD91038

SDD32831

VXC20469

**Fig. SI6.** (A) Phylogenetic analysis of BLC23O with other *Bacillus* C23Os. (B) Sequence comparison of BLC23O with selected *Bacillus* C23Os. Details of operations for (A) were described in Fig. 5 legend and for (B) in Fig. 4 legend.

**Table SI1.** Metal content analysis of as-purified BLC23O. The as-purified BLC23O was analyzed by Eurofins Environment Testing Canada Inc. (Ottawa, Ontario, Canada) using EPA 200.8 (ICP-MS) method. The control sample contained buffer alone + 1mM Mn (II) and the as-purified enzyme was prepared in 100mM Tris-HCl, pH 7.5 with a total protein content of 2.6 mg. An amount that showed significant enzyme activities in the presence of Mn (II).

| Metal  Analyte | Control (buffer + Mn) | As purified BLC23O in buffer | MRL  (Method reporting limit) | Units |
| --- | --- | --- | --- | --- |
| Silver (Ag) | 0.0007 | 0.0007 | <0.0001 | mg/L |
| Aluminum (Al) | <0.01 | <0.01 | <0.01 | mg/L |
| Boron (total) (B) | <0.01 | <0.01 | <0.01 | mg/L |
| Barium (Ba) | <0.01 | <0.01 | <0.01 | mg/L |
| Beryllium (Be) | <0.0005 | <0.0005 | <0.0005 | mg/L |
| Cadmium (Cd) | <0.0001 | <0.0001 | <0.0001 | mg/L |
| Cobalt (Co) | <0.0002 | <0.0002 | <0.0002 | mg/L |
| Chromium (Total) (Cr) | <0.001 | <0.001 | <0.001 | mg/L |
| Copper (Cu) | <0.001 | <0.001 | <0.001 | mg/L |
| **Iron (Fe)** | **<0.03** | **<0.03** | <0.03 | mg/L |
| **Manganese (Mn)** | **0.17** | **<0.01** | <0.01 | mg/L |
| Molybdenum  (Mo) | <0.005 | <0.005 | <0.005 | mg/L |
| Nickel (Ni) | <0.005 | <0.005 | <0.005 | mg/L |
| Lead (Pb) | <0.001 | <0.001 | <0.001 | mg/L |
| Silicon (Si) | 0.1 | 0.1 | <0.1 | mg/L |
| Strontium (Sr) | <0.001 | <0.001 | <0.001 | mg/L |
| Titanium (Ti) | <0.01 | <0.01 | <0.01 | mg/L |
| Thallium (Tl) | <0.0001 | <0.0001 | <0.0001 | mg/L |
| Vanadium (V) | <0.001 | <0.001 | <0.001 | mg/L |
| Zinc (ZN) | <0.01 | <0.01 | <0.01 | mg/L |

**References**

1 Saitou, N. & Nei, M. The neighbor-joining method: a new method for reconstructing phylogenetic trees. *Molecular biology and evolution* **4**, 406-425 (1987).

2 Zuckerkandl, E. & Pauling, L. in *Evolving Genes and Proteins* (eds Vernon Bryson & Henry J. Vogel) 97-166 (Academic Press, 1965).

3 Kumar, S., Stecher, G., Li, M., Knyaz, C. & Tamura, K. MEGA X: Molecular Evolutionary Genetics Analysis across Computing Platforms. *Molecular Biology and Evolution* **35**, 1547-1549, doi:10.1093/molbev/msy096 (2018).
